# Transfer RNAs Mediate the Rapid Adaptation of *Escherichia coli* to Oxidative Stress

**DOI:** 10.1371/journal.pgen.1005302

**Published:** 2015-06-19

**Authors:** Jiayong Zhong, Chuanle Xiao, Wei Gu, Gaofei Du, Xuesong Sun, Qing-Yu He, Gong Zhang

**Affiliations:** 1 Key Laboratory of Functional Protein Research of Guangdong Higher Education Institutes, Institute of Life and Health Engineering, College of Life Science and Technology, Jinan University, Guangzhou, China; 2 State Key Laboratory of Ophthalmology, Zhongshan Ophthalmic Center, Sun Yat-Sen University, Guangzhou, China; The Ohio State University, UNITED STATES

## Abstract

Translational systems can respond promptly to sudden environmental changes to provide rapid adaptations to environmental stress. Unlike the well-studied translational responses to oxidative stress in eukaryotic systems, little is known regarding how prokaryotes respond rapidly to oxidative stress in terms of translation. In this study, we measured protein synthesis from the entire *Escherichia coli* proteome and found that protein synthesis was severely slowed down under oxidative stress. With unchanged translation initiation, this slowdown was caused by decreased translation elongation speed. We further confirmed by tRNA sequencing and qRT-PCR that this deceleration was caused by a global, enzymatic downregulation of almost all tRNA species shortly after exposure to oxidative agents. Elevation in tRNA levels accelerated translation and protected *E*. *coli* against oxidative stress caused by hydrogen peroxide and the antibiotic ciprofloxacin. Our results showed that the global regulation of tRNAs mediates the rapid adjustment of the *E*. *coli* translation system for prompt adaptation to oxidative stress.

## Author Summary

All organisms need to respond quickly to sudden environmental changes. Translational regulation can occur in response to environmental stresses within minutes, which is much faster than transcriptional regulation, and thus normally provides immediate adaptation. Eukaryotic cells can manipulate their tRNA molecules, mainly in a reversible manner, to suppress translation. Here, we showed for the first time that bacteria respond to oxidative stress by adjusting the translational system in a manner that differs from that of eukaryotes. The bacteria nonspecifically, irreversibly, and enzymatically degrade tRNAs to block protein synthesis. Interestingly, we showed that elevated tRNA concentrations lead to opposing effects by causing increased protein aggregation, which impairs fitness under normal conditions but facilitates adaptation under oxidative stress, including that caused by antibiotics. Our results provide a new understanding of the role of global adjustments to the entire translation system during stress adaptation in bacteria. This mechanism may also be involved in the development of antibiotic resistance in bacteria.

## Introduction

Reactive oxygen species (ROS), such as hydrogen peroxide (H_2_O_2_), the hydroxyl radical (·OH), and superoxide (O_2_^-^), are mainly generated as byproducts of the respiratory chain or introduced on exposure to a hazardous environment [1]. These ROSs can damage proteins and nucleic acids by oxidation, leading to cellular oxidative stress (reviewed in [1–3]). To counteract oxidative stress, bacteria have evolved diverse systems, such as the *OxyR* system and *SoxRS* systems, to activate the transcription of a series of enzymes including superoxide dismutases (reviewed in [3]). However, the transcription of the stress-response genes requires 10 min to reach maximal production levels, and protein translation requires additional time for synthesis [4,5], which takes ~20–30 min to take effect in bacteria and 45 min in yeast (reviewed in [6]). Thus, cells need an alternative mechanism(s) to response to environmental stresses within minutes, i.e., at the translation level (reviewed in [7]).

Translational regulation under oxidative stress has been intensively studied in eukaryotic cells. Early studies suggested that translation is globally inhibited in 5 min in *Saccharomyces cerevisiae* cells [8]. This process is mediated by specific tRNA and rRNA cleavage [9,10]. The same phenomenon has also been observed in mammalian cell lines [11–13]. Cleavage of tRNAs leads to the formation of small RNA fragments that can repress translation initiation and can regulate cellular functions, such as proliferation [14,15]. However, recent studies have provided increasing evidence that a considerable fraction of genes is more actively translated under oxidative stress in eukaryotic cells. In fission yeast, 26 genes were translationally upregulated in 15 min, and 191 genes were translationally upregulated in 60 min [5]. Results from a time-resolved transcriptome and proteome study in *S*. *cerevisiae* revealed that >80% of proteins diverged from their mRNA expression profiles, suggesting a widespread translational control mechanism involving both upregulation and downregulation. Approximately 25% of the proteins were upregulated with their mRNA expression levels nearly unchanged[16]. The tRNA^Leu(CAA)^ hypermodified at the wobble position increases the translation of TTG codons after H_2_O_2_ exposure and thus enhanced the protein expression of TTG-enriched genes [17].

In contrast, little is known about translational regulation in response to oxidative stress in prokaryotes, although the available data have provided clues suggesting that this response differs substantially from that of eukaryotes. For example, specific endoribonucleases such as colicin and PrrC specifically cleave tRNA into 2 fragments at anticodon loop in prokaryotes; however, this phenomenon is not as universal in prokaryotes as it is in eukaryotes, occurring only under specific conditions with a select few tRNAs such as tRNA-Arg(ICG) [18] and tRNA-Lys [19,20].

Recent “omics” reports investigating alterations in bacteria have been focused on steady-state conditions. The translational fidelity is slightly lowered due to increased tRNA mischarging [21]. However, the mild effects of mischarging can be easily overcome by an adequate supply of amino acids [21]. Modified *Escherichia coli* strains can adapt to their oxidative cytoplasmic environments to yield growth rates similar to those of the wild-type [22]. Such “oxidative” *E*. *coli* strains have been used in industrial applications to produce recombinant proteins that contain disulfide bonds, showing its strong fitness (reviewed in [23]). The chaperone system (e.g., DnaK, Tig, and GroEL) remains nearly constant or only marginally decreased under oxidative stress, indicating that oxidative stress does not lead to as much protein unfolding or damage as the heat stress[24], which is in sharp contrast to the effects observed following heat shock [25,26]. Although it has been proposed that polynucleotide phosphorylase may globally degrade oxidized RNAs containing 8-oxo-G to protect *E*. *coli* from oxidative damage [27], results from multiple transcriptome and proteome studies have shown that transcriptomes and proteomes are nearly unchanged by such activity. For example, only 6 differentially expressed proteins were found in *E*. *coli* when exposed to oxidative stress, only 2 of which are transcriptionally regulated in response to oxidative stress [24]. Similar results were found in *Bacillus subtilis* [28]. Notably, ~200 differentially expressed mRNAs were detected in *B*. *subtilis* under oxidative stress, but only 16 of the corresponding proteins showed differential expression. Discrepancies between the numbers of differentially expressed genes and proteins suggest that a remarkable translational regulation process occurs in response to oxidative stress in prokaryotes. However, a general translational response remains elusive.

The protein synthesis rate is a critical index for representing the translational efficiency, especially for a nearly steady-state proteome, and can be measured using stable isotope labeling by amino acids in cell culture mass spectrometry (SILAC-MS) [29–31]. Briefly, cells are transferred into SILAC media containing amino acids (usually lysine or arginine) labeled with heavy isotopes, and the newly synthesized proteins are labeled with the heavy isotopes. The ratio of heavy to light peptides can be accurately measured by mass spectrometry (reviewed in [32]). Thus, the protein synthesis rate can be deduced by monitoring the production of heavy peptides in a time-course assay. This strategy works well for higher eukaryotic systems, but is not applicable in prokaryotes due to their complex metabolic pathways that convert labeled lysine or arginine residues to other amino acids, which deviates from the principle of metabolic labeling [33]. Metabolic labeling of all nitrogen atoms with ^15^N can label entire proteins very effectively (>99%) [34], but creates complex *m/z* shifts due to the large number of nitrogen atoms present in peptides, which severely challenges data interpretation. Therefore, poor identification is expected, and the turnover of only 40 proteins in the green alga *Ostreococcus tauri* could be analyzed using this method [35]. To bypass this disadvantage, Maier *et al*. used 30 external isotope-labeled peptides as references and determined half-lives of 231 proteins in *Mycoplasma pneumoniae* [26], which is a more effective approach than those used in previous studies, but is relatively complicated.

In this study, we improved upon our previously developed ProVerB algorithm [36] to identify and quantify ^15^N-metabolically labeled proteins in time-course assays using MS. Thus, we could measure protein synthesis in *E*. *coli* under normal conditions and oxidative stress at the proteome-wide level. Surprisingly, we found that translation elongation nearly halted, minutes after the oxidant was applied. We detected no specific tRNA and rRNA degradation, but levels of full-length tRNAs were rapidly reduced. Interestingly, eleva-ted tRNA concentrations promoted the fitness of *E*. *coli* under oxidative stress, making it more resistant to oxidation-generating antibiotics. These results depict a unique tRNA-centric scheme of global translational responses during oxidative stress in prokaryotes, which is different from the regulatory mechanisms used in eukaryotes.

## Results

### Optimal condition of oxidative stress

**Fig 1:**
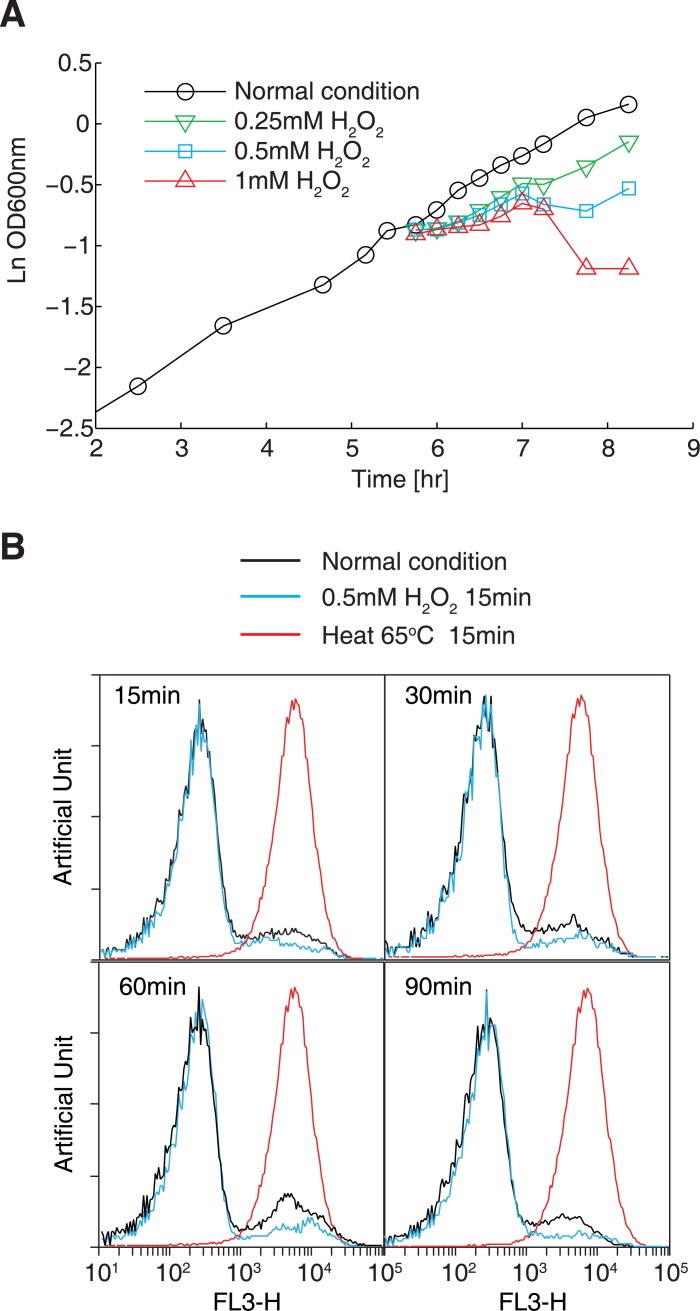
Optimization of the oxidative stress conditions. (A) Cell growth in the presence of different concentrations of H_2_O_2_. *E*. *coli* BL21(DE3) cells were cultivated in M9 minimal medium. Cell growth in the presence of 0.25–1 mM H_2_O_2_ was monitored by measuring OD_600_ values. (B) Cell viability assay using propidium iodide staining and flow cytometry. Histograms showing cells exposed to 0.5 mM H_2_O_2_ for 15, 30, 60, or 90 min were obtained (cyan lines). Cells grown under normal condition was used as a positive control (black line), and cells killed by incubation at 65°C for 15 min were used as a negative control (red line).

To determine optimal conditions for inducing oxidative stress, we measured the growth of the *E*. *coli* BL21 strain in M9 minimal medium with various concentrations of H_2_O_2_, ranging from 0.2 mM to 2 mM. Exposing cells to 0.5 mM H_2_O_2_ resulted in marked transient growth arrest (10-fold decrease in the growth rate), while higher concentrations (1 mM and 2 mM) led to considerable cell death (Fig 1A). This concentration is comparable to that used in previous studies [37,38]. In parallel, we performed propidium iodide (PI) staining and flow cytometry to examine the degree of cell death caused by oxidative reagents. Treating cells with 0.5 mM H_2_O_2_ resulted in a nearly identical profile of low-stained cells (representing live cells) under normal conditions after at least 90 min following incubation with H_2_O_2_ (Fig 1B). In contrast, the cells killed at 65°C for 15 min showed a single peak with high PI staining. These results showed that treating cells with 0.5 mM H_2_O_2_ does not cause detectable cell death.

### Proteome synthesis measured by ^15^N metabolic labeling

**Fig 2:**
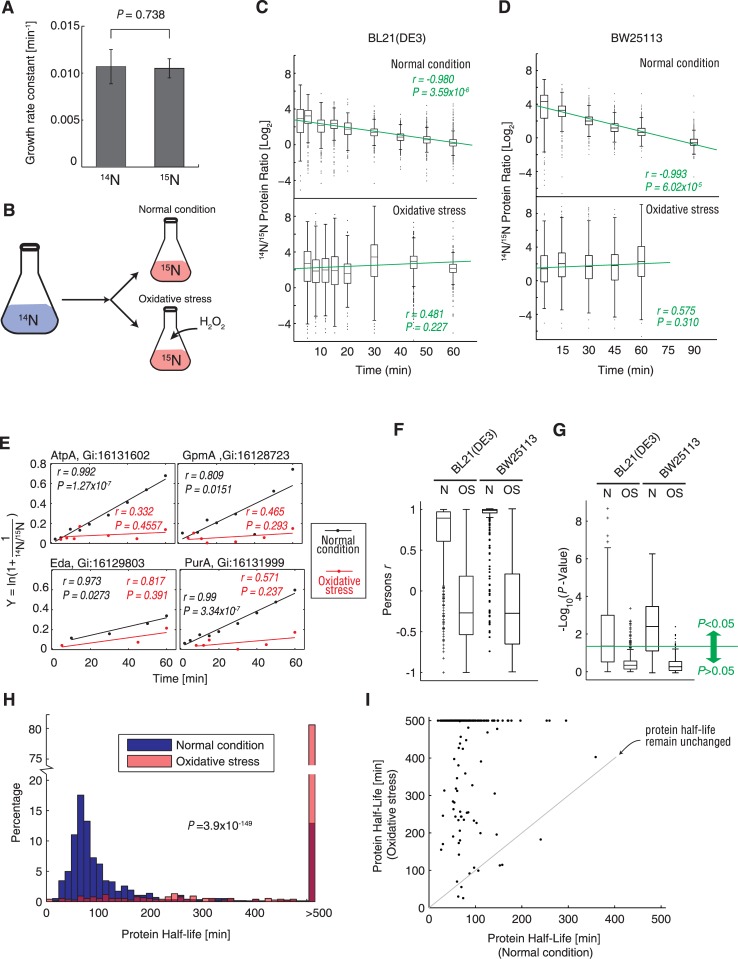
Protein synthesis measurement under normal condition and oxidative stress using ^15^N metabolic labeling and mass spectrometry. (A) Growth rate constant of *E*. *coli* BL21(DE3) cells grown under normal conditions in M9 minimal medium containing ^14^NH_4_Cl (^14^N) or ^15^NH_4_Cl (^15^N). (B) Experimental design for pulse labeling with ^15^N under normal and oxidative stress conditions. (C) Bacterial cells were transferred to M9 medium containing ^15^N, after which the ^14^N/^15^N ratio of the *E*. *coli* BL21(DE3) proteome was determined by mass spectrometry under normal conditions (upper plot) and oxidative stress induced by exposure to 0.5 mM H_2_O_2_ (lower plot). The green line indicates the best linear fit of the dataset. The Pearson *r* correlation coefficient and *P*-value of the fit are indicated in green text. (D) The same experiment represented in (C) was repeated using the wild-type *E*. *coli* BW25113 strain. (E) Examples showing changes in the ^14^N/^15^N ratio of 4 randomly selected proteins (AtpA, GpmA, Eda, and PurA) in BL21(DE3) cells over time. The fit was determined according to Eq 6 (Materials and Methods section). The slope of the linear fit is the synthesis rate constant (*k*_syn_). The black dots indicate the actual data points following protein quantification under normal condition, while the red dots indicate the data points measured under oxidative stress. Linear fits were determined for each dataset, and the Pearson correlation coefficients (*r*) are indicated in the diagram. (F) The Pearson correlation coefficients (*r*) values of all proteins were fitted using Eq 6, under normal condition and oxidative stress. Data generated using the BL21(DE3) and wild-type BW25113 strains are shown. (G) *P* values (in -log_10_ scale) of linear fits of all proteins using Eq 6 under normal and oxidative stress conditions. The green line denotes the significance threshold (*P* = 0.05). The data generated using the BL21(DE3) and wild-type BW25113 strains are shown. (H) Histograms of protein half-lives under normal (blue) and oxidative stress (red) conditions. (I) Comparison of protein half-lives measured under normal and oxidative stress conditions. The grey line is at a 45° angle, which demarks the positions where the protein half-lives were unchanged. Half-lives longer than 500 min are shown as 500 min.

To study the translational response of *E*. *coli* cells under oxidative stress, we investigated protein synthesis (amino acids incorporation) under normal and oxidative stress conditions. To achieve accurate quantification, ^15^N metabolic labeling was used, as SILAC labeling is not applicable for bacterial studies [33]. However, it has been reported previously that ^15^N metabolic labeling can cause a significant decrease in growth rates and significant alterations in metabolite production in the *E*. *coli* BL21star strain. These findings indicate that the metabolism is remarkably changed just because of the single extra neutron in heavy nitrogen atoms and that this method can thus introduce bias in analysis of protein synthesis [39]. To examine this effect in our system, we measured the growth curve of the *E*. *coli* BL21(DE3) strain in M9 minimal medium with ^14^NH_4_Cl or ^15^NH_4_Cl as the only nitrogen source. Interestingly, we could not detect any significant differences in growth rates during log-phase growth using these two isotopes (*P* = 0.738, two-tailed Student *t*-test; Fig 2A). This indicated that our ^15^N metabolic-labeling protocol did not create any growth defects and that it can be used for analyzing protein synthesis.

To overcome the drawbacks of traditional MS identification and quantification algorithms in the context of ^15^N metabolic labeling, we used our ProVerB algorithm [36], which is capable of handling complex *m/z* shifts caused by ^15^N metabolic labeling, as described in the Materials and Methods section. Peptides were identified from spectra matches to our tandem MS/MS spectra. Two spectral matches are shown in S1 Fig. These spectra demonstrated the reliable identification of peptides, even when noise spectra peaks were present. The ^14^N/^15^N ratios of peptides were determined based on the peak areas of the MS1 scans. Using a stringent criterion of the false discovery rate (FDR) <1% at the protein level, we quantified ~300–1000 proteins from single *E*. *coli* soluble protein samples (quantifications shown in S1 Table), which is markedly higher than results obtained using previous algorithms. This allowed us to assess the protein-synthesis rate of *E*. *coli* on a proteome-wide scale in high resolution for the first time.

We cultured *E*. *coli* BL21(DE3) cells until the optical density at 600 nm (OD_600_) reached 0.4 in ^14^N M9 minimal medium and then quickly changed the medium to ^15^N labeled minimal medium, with or without 0.5 mM H_2_O_2_. Next, we collected the cells at various time points and analyzed their proteomes by MS (Fig 2B). We quantified the ^14^N/^15^N ratios for each protein at each time point with our new algorithm. Without oxidative stress, ^15^N-labeled proteins were continuously synthesized and replaced the ^14^N proteins, resulting in a constant decrease of the ^14^N/^15^N ratio over time (Fig 2C, upper plot), indicating that the ^15^N-labeled proteins gradually substituted for their ^14^N counterparts. This finding was confirmed by performing linear regression of the data points, which showed a very significant and negative slope (Pearson correlation coefficient *r* = -0.980, *P* = 3.59 × 10^-6^; Fig 2C, upper plot). In sharp contrast, the ^14^N/^15^N ratio of the proteome showed negligible differences over time under oxidative stress (insignificant linear fit with *P* = 0.227; Fig 2C, lower plot). S2 Fig shows the MS1 scans obtained for 2 peptides after 60 min growth under oxidative stress and normal conditions, as examples. Under normal condition, the quantity of the newly synthesized ^15^N peptides exceeded their ^14^N counterparts (S2A Fig). In contrast, under oxidative stress, the quantities of newly synthesized ^15^N peptides were at least one order of magnitude lower than the corresponding ^14^N peptides (S2B Fig). These results indicate that protein synthesis is almost completely blocked under oxidative stress. We performed the same proteomic measurements in the wild-type *E*. *coli* BW25113 strain and reproduced the same trend (Fig 2D).

**Fig 3:**
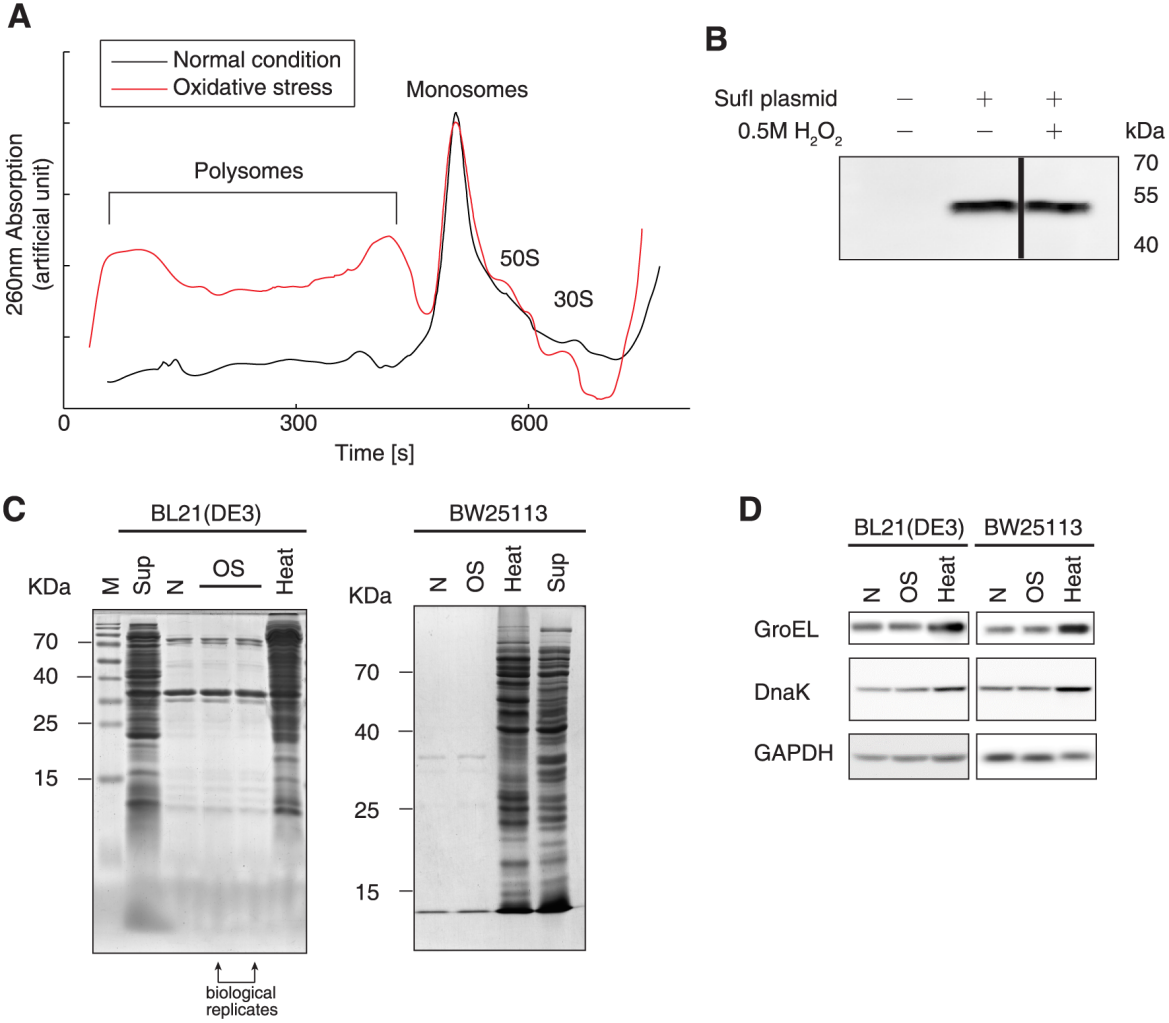
Protein biogenesis under oxidative stress. (A) Polysome profiles under normal and oxidative stress conditions. (B) SufI synthesis in an *in vitro* translation system, with or without oxidative stress. The SufI gene fused with a C-terminal His_6_-tag sequence constructed in a pET-28b plasmid (Novagen) was used as a template in an *in vitro* translation reaction. The protein product was detected by western blotting with an anti-His_6_ antibody. (C) Detergent-insoluble proteins under normal (N) and oxidative stress (OS) conditions. Insoluble proteins extracted from *E*. *coli* BL21(DE3) and wild-type BW25113 cells grown at 47°C for 10 min were used as positive controls (Heat). Soluble proteins (Sup) were loaded on the same gel as controls. (D) Western blot analysis of the molecular chaperones GroEL and DnaK in *E*. *coli* BL21(DE3) and wild-type BW25113 cells grown under normal (N) conditions, oxidative stress (OS) condition, or heat stress (heat) at 47°C for 30 min.

To validate these findings, we calculated the synthesis rate constant (*k*_syn_) of those proteins that were quantified in at least 3 time points, using Eq 6. The linear fits for 4 randomly selected proteins are demonstrated in Fig 3E, showing that the *k*_syn_ (equal to the slope) of most proteins under oxidative stress was much lower than that observed under normal conditions. The 4 fits under normal conditions were all significant (*P* < 0.05), while none of the fits under oxidative stress were significant (*P* = ~0.237–0.457), indicating that we could not reliably detect the protein synthesis rate of these proteins, i.e., the protein synthesis was blocked. The linear fits (Eq 6) of most proteins yielded Pearson correlation coefficient (*r*) >0.6 in BL21(DE3) cells, and approached 1 in the wild-type BW25113 strain, indicating a good linear fit. In sharp contrast, the Pearson *r* values of most proteins were <0.2 in both strains (Fig 2F). Moreover, more than half of the fits under normal conditions were statistically significant (*P* < 0.05), while 95% of the fits obtained under oxidative stress were insignificant (*P* > 0.05), indicating that oxidative stress caused an overall shutdown in proteome synthesis in both strains (Fig 2G).

We then calculated the half-lives for each protein using Eq 7. The average half-life of the proteome under normal conditions was 100.38 min, with >69% of the individual proteins having a half-life <100 min (Fig 2H). In sharp contrast, >80% of the proteins showed a half-life exceeding 500 min under oxidative stress, which significantly deviated from the half-life distributions under normal conditions (*P* = 3.9 × 10^-149^, Kolmogorov-Smirnov test; Fig 2H). Half-lives of >500 min could not be reliably detected (*P* > 0.05) using the linear fit of Eq 6. The vast majority of proteins were regenerated much more slowly under oxidative stress (Fig 2I). In sum, these data revealed that protein synthesis was inhibited or nearly abolished under conditions of oxidative stress.

### Oxidative stress slows down translation elongation, but does not affect translation initiation

To investigate whether inhibited protein synthesis occurs in the initiation or elongation phase, we measured polysome profiles in *E*. *coli* grown with and without oxidative stress. Cells under oxidative stress showed remarkably higher fractions of polysomes than observed under normal conditions, indicating that the translation initiation frequency may be unchanged, but that elongation was considerably decelerated (Fig 3A). This finding agreed with our previous data showing that an unchanged translation initiation and faster elongation speed results in a decrease in the polysome fraction [40].

To further validate this hypothesis, we performed cell-free translation of the *E*. *coli* protein SufI expressed with a C-terminal His_6_-tag, in the presence or absence of 0.5 mM H_2_O_2_. The reaction mixtures contained only the target gene and the necessary components required for translation, and no protease or ribonuclease was present. Therefore, the protein production rate is determined solely by translation initiation in this system. The SufI protein synthesized in the cell-free translation system was detected by western blot analysis, which showed that H_2_O_2_ incubation did not inhibit *in vitro* protein production (Fig 3B). This suggested that translation initiation is not hindered by oxidative reagents, i.e., the oxidative reagents used in this study did not cause significant oxidative damage to the proteins.

**Fig 4:**
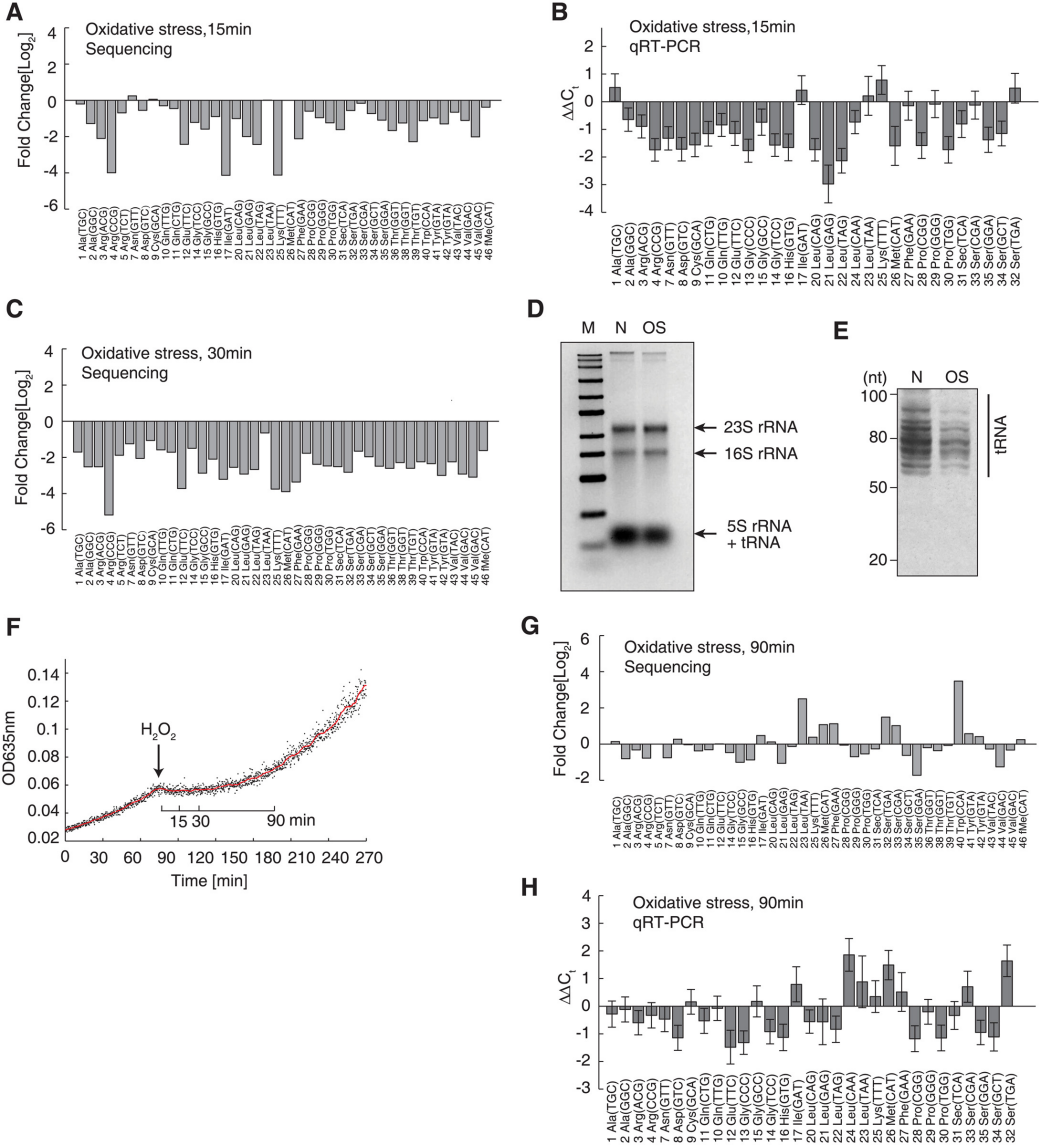
Decrease of full-length tRNAs under oxidative stress. The tRNAs are displayed in alphabetical order and named according to their amino acids and anticodons. (A) Decreased tRNA levels following 15 min of H_2_O_2_-induced oxidative stress, relative to tRNA levels observed under normal conditions. The data were generated by next-generation sequencing. (B) qRT-PCR validation of the tRNA decreases induced by 15 min of oxidative stress, using independent biological replicates. The data are shown as the mean ± SD. (C) Decreased tRNA levels following 30 min of OS, measured by next-generation sequencing. (D) Agarose gel electrophoresis of the total RNA extracted from the same number of cells grown under normal conditions (N) or under 15 min of oxidative stress (OS). (E) Polyacrylamide gel electrophoresis of the sample represented in panel (D) to resolve the tRNAs. The size range of tRNAs is indicated. (F) Growth curves before and after the application of oxidative stress. The red line shows the moving average of the OD_635_ observed over 5 min. (G) The tRNA concentration after 90 min of oxidative stress, after which the bacteria had fully adapted to the stress and growth was resumed. (H) qRT-PCR validation of the results shown in panel (G), using an independent biological replicate. The data are shown as the mean ± SD.

To test this postulation, we examined protein aggregation, as oxidative damage to proteins results in protein misfolding and aggregation in cells [41]. Under normal conditions, we observed a pattern of protein aggregation (Fig 3C) that was in agreement with results from a previous report [40]. Under the oxidative stress condition used in this study, we did not observe increased protein aggregation in either *E*. *coli* strain tested (Fig 3C). As a positive control, we showed that heat stress at 47°C led to severe protein misfolding and aggregation. We also tested the molecular chaperones GroEL and DnaK, which serve as markers for heat shock and protein aggregation responses. The levels of these chaperones remained approximately equal under normal and oxidative stress conditions, but were remarkably elevated at a high temperature (Fig 4D). These results suggested that oxidative stress did not generate massive protein aggregation.

Given that the proteome was not damaged, we hypothesized that the inhibition of translation elongation may have been caused by the downregulation of tRNA abundances *in vivo*.

### Full-length tRNA levels are globally downregulated under oxidative stress *in vivo*

To study *in vivo* variations in tRNA abundances upon oxidative stress, we quantitatively analyzed the tRNA pool by next-generation sequencing and qRT-PCR. We added an *in vitro* transcribed single-stranded RNA as a spike-in RNA. This spike-in contained a 3′-CCA tail like tRNA and deviates by least 3 nucleotides from any *E*. *coli* tRNA (S3 Fig). We obtained ~8–10 million sequencing reads for cells under normal and oxidative conditions. A total of 1.6~2.0 million reads were aligned to 47 tRNA species using a modified Smith–Waterman algorithm, which considered modified bases according to the Modomics database and allowed for a maximum of 3 errors [42,43]. All 47 tRNA species were detected under both conditions with high reproducibility (*R* = 0.998, independent biological replicates; S4A Fig), except for 2 methionine tRNAs (S2 Table). The lack of detection of the 2 methionine tRNAs may have been due to the higher number of mismatches occurring in the sequencing reads. After normalization to the spike-in RNA, we detected a global downregulation of full-length tRNA (on average, a 2.47-fold decrease) 15 min after the application of oxidative stress (Fig 4A). This trend was in general validated by qRT-PCR performed using an independent biological replicate, with which 38 tRNA species were successfully and specifically amplified (Fig 4B). Notably, the qRT-PCR results obtained with independent biological replicates showed very high reproducibility (*R* ≥ 0.99; S4B Fig). These tRNA levels were further decreased after 30 min of oxidative stress (Fig 4C). With ribosomal RNA levels remaining constant under oxidative stress, the decrease in low molecular weight RNAs could be clearly seen (Fig 4D), indicating a decrease in tRNA levels. This global decrease in tRNA was then visually confirmed by polyacrylamide gel electrophoresis, which can resolve tRNAs into multiple bands (Fig 4E). To rule out possible technical artifacts, we collected the cells 90 min after the application of oxidative stress, during which time the cells adapted the new environment and cell growth was resumed (Fig 4F), implying that protein synthesis was restored. Both next-generation sequencing and qRT-PCR showed that tRNA production was restored to normal or even slightly higher levels (Fig 4G and 4H). To rule out a potential bias occurring during quantitative reverse-transcription reactions associated with the CCA-specific reverse-transcription method, we used a traditional method with random hexamers to prime the reverse-transcription reactions, which is not 3’-CCA specific [44,45]. We could reproduce the global and significant downregulation of tRNAs 15 min after oxidative stress, which was reversed after 90 min (S5 Fig). To demonstrate that this trend was not strain-specific, we reproduced the effect of global tRNA downregulation after 15 min of oxidative stress (with reversal after 90 min) in the wild-type *E*. *coli* BW25113 strain (S6 Fig). These results showed that the abundances of full-length tRNAs were indeed decreased under oxidative stress, and thus inhibited the translation elongation.

### Enzymatic and nonspecific tRNA degradation decreases full-length tRNA concentrations under oxidative stress

To determine how the full-length tRNAs were degraded, we first examined whether tRNAs were specifically cleaved into halves in *E*. *coli*, similar to the phenomenon observed in eukaryotes [9]. However, no bands or smears could be detected at ~30–40 nt in a polyacrylamide gel, suggesting that specific tRNA degradation did not occur in *E*. *coli* under oxidative stress (Fig 4E). The lack of smaller bands or smear below the tRNA bands also suggested that RNAs in general was not degraded.

**Fig 5:**
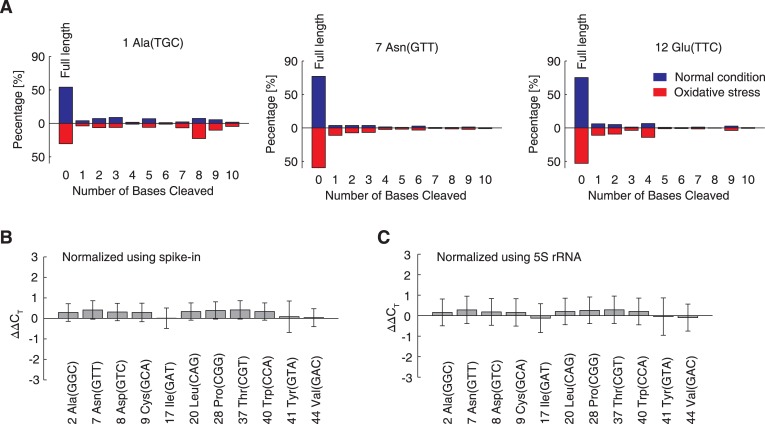
Under oxidative stress, tRNAs are degraded *in vivo*, but not in the cell-free *in vitro* translation system. (A) Percentages of cleaved tRNA reads. X-axes indicate the number of bases cleaved from the 3′-termini. The Y-axes denote the fraction of such tRNA reads among all tRNA reads relative to each specific tRNA species. Three tRNAs are shown as examples. The observed distributions of the cleavage lengths were compared between normal (blue bars) and oxidative stress conditions (red bars). (B,C) The degradation of full-length tRNA in *in vitro* translation system. Eleven tRNAs were randomly selected and quantified by qRT-PCR. The difference observed between the oxidative stress and normal conditions are expressed in terms of ΔΔC_T_ values, normalized using spike-in RNA (B) or 5S rRNA (C). The data are shown as the mean ± SD.

We then investigated the non-full-length tRNA reads in the sequencing datasets. Three tRNAs, namely, 1-Ala(TGC), 7-Asn(GTT), and 12-Glu(TTC), possess tandem CCA sequences at their 3′-end (i.e., CCACCA-3′). Therefore, if the 3′-CCA sequences were specifically cleaved, the remaining sequence could be detected by our method. However, in contrast to eukaryotes, in which the 3′-CCA tails can be reversibly cleaved to temporarily block translation [13], no accumulation of 3′-CCA cleavage intermediates was detected in *E*. *coli* cells under oxidative stress. Reads that were ~1–10 nucleotides shorter than the full-length sequence were detected, and the versions without 3′-CCA were not the most prevalent species for most tRNAs (Fig 5A). These results indicated that tRNA degradation under oxidative stress is nonspecific and irreversible, which stands in contrast to the situation observed in eukaryotic cells.

The next open question was whether the observed nonspecific tRNA degradation relied upon cellular enzymes like RNases. To address this question, we added 0.5 mM H_2_O_2_ in the *E*. *coli* cell-free *in vitro* translation system with an RNase inhibitor and quantified 11 tRNAs by qRT-PCR. Compared to the *in vitro* translation results found without H_2_O_2_, none of the tRNAs in the oxidative environment were degraded; their quantities remained unchanged (Fig 5B and 5C). This conclusion was validated when qRT-PCR results were normalized according to the quantity of spike-in tRNA (without nucleotide modifications, Fig 5B) and 5S rRNA (with nucleotide modifications, Fig 5C), showing that the oxidation of the modified nucleotides did not affect the cDNA-based quantitation methods. Therefore, the nonspecific degradation observed *in vivo* under oxidative stress is dependent on cellular enzymes.

### Elevated tRNA levels protects *E*. *coli* against oxidative stress

**Fig 6:**
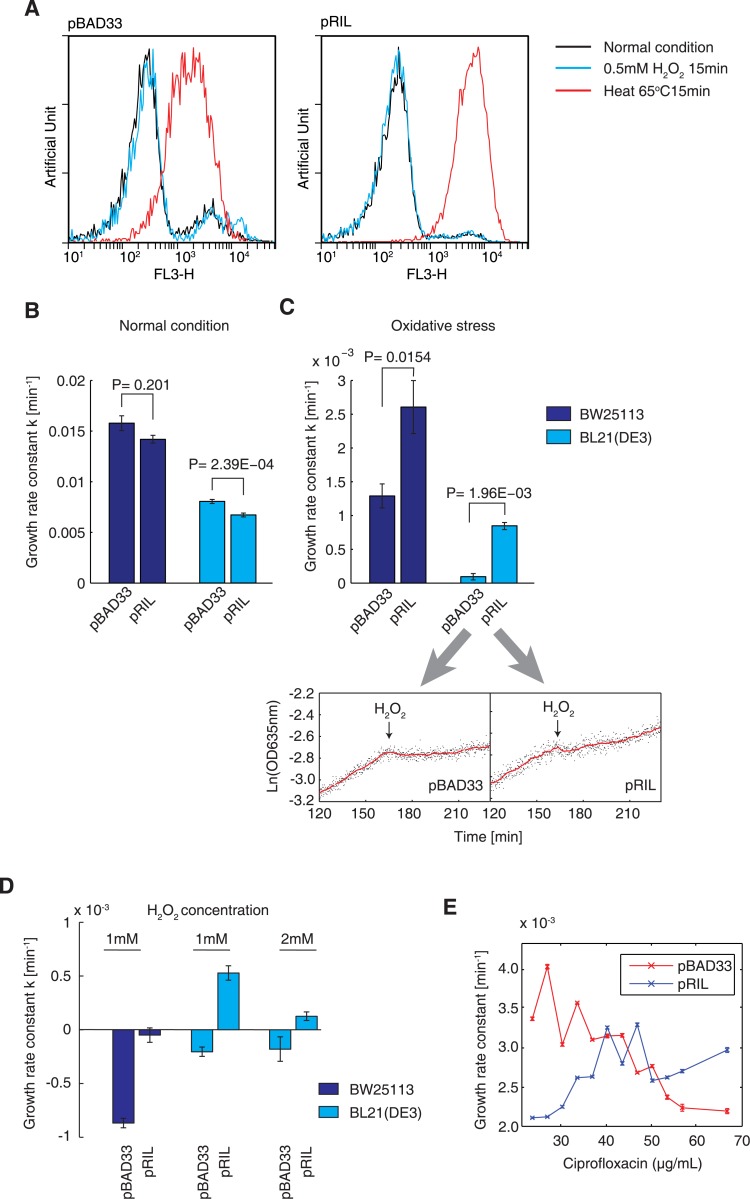
Higher tRNA concentrations improve adaptation under oxidative stress. (A) Cell viability testing results obtained using the PI staining method. *E*. *coli* BL21(DE3) cells carrying the empty vector pBAD33 and the pRIL plasmid were tested. A histogram showing cells under 0.5 mM H_2_O_2_-induced oxidative stress for 15 min was prepared. Cells grown under normal conditions were used as a positive control, and cells killed by incubation at 65°C for 15 min were used as a negative control. (B) Growth rate constants of *E*. *coli* BW25113 and BL21(DE3) carrying the pBAD33 and pRIL plasmids, under normal condition. The values shown are the mean ± SD. (C) Growth rate constants of *E*. *coli* BW25113 and BL21(DE3) carrying the pBAD33 and pRIL plasmids, under oxidative stress. The actual growth curves of BL21(DE3) carrying these two plasmids are shown respectively. The red line is the moving average observed over 5 min. (D) Cell growth-rate constant under harsh oxidative stress (~1–2 mM H_2_O_2_). A negative growth rate constant represents the occurrence of cell death. The data shown are the mean ± SD. (E) The growth-rate constant of *E*. *coli* BW25113 cells carrying the pBAD33 and pRIL plasmid, cultured in the presence of different concentrations of ciprofloxacin.

Translation elongation was remarkably inhibited due to nonspecific tRNA degradation. Therefore, we posited that excess tRNAs may rescue translation elongation under oxidative stress. To test this hypothesis, we elevated the levels of low-abundance tRNAs in the wild-type *E*. *coli* BW25113 strain and the BL21(DE3) strain by transforming them with the pRIL plasmid. This plasmid was isolated from the *E*. *coli* BL21(DE3)-CodonPlus RIL (BL21-RIL) strain, which carries extra copies of tRNAs that decode AGA, AGG, AUA, and CUA codons and, thus, can significantly accelerate translation elongation [40,45]. Control *E*. *coli* cells were transformed with the empty pBAD33 vector, which is chloramphenicol resistant like pRIL, to exclude any potential influence caused by chloramphenicol selection [40]. The oxidative environment did not cause elevated cell death in BL21-RIL cells or control cells without tRNA supplementation (Fig 6A). The pRIL plasmid speed up the translation, resulting in augmented protein misfolding and aggregation [40], thus significantly impairing the fitness and growth rate, compared to control cells (*P* = 2.39 × 10^-4^, two-tailed Student *t*-test; Fig 6B). In the wild-type BW25113 strain, tRNA supplementation also slightly decreased the growth rate, but this difference was not significant (*P* = 0.201, two-tailed Student *t*-test; Fig 6B), perhaps because the wild-type *E*. *coli* strains expresses the OmpT and Lon proteases and can degrade the aggregated misfolded proteins more efficiently than the BL21 strain. In contrast, when oxidative stress was applied, both the wild-type BW25113 and the BL21 cells with augmented tRNAs grew significantly faster than the control cells (*P =* 0.0154 and 1.96 × 10^-3^, respectively, two-tailed Student *t*-test; Fig 6C), which supported our hypothesis.

We then posited that the protective effect afforded by tRNA upregulation increases the survival of *E*. *coli* under harsh oxidative stress. Therefore, we increased the H_2_O_2_ concentration to ~1–2 mM. In the presence of ~1–2 mM H_2_O_2_, the control BL21 cells died (Fig 6D), in agreement with the results shown in Fig 1A. In contrast, the tRNA-supplemented BL21-RIL cells continued to grow, although at a slower rate (Fig 6D). In the presence of 1 mM H_2_O_2_, the BW25113 control cells died rapidly, while the tRNA-supplemented BW25113-RIL cells survived (Fig 6D). These results showed that elevated tRNA concentrations alone could increase the resistance of *E*. *coli* cells to harsh oxidative stress.

To validate the phenomenon of tRNA-mediated resistance using another oxidative reagent, we incubated *E*. *coli* cells with ciprofloxacin, an antibiotic that induces extensive intracellular oxidative radical formation in prokaryotes [46,47], and examined whether increased tRNA concentrations improves the fitness of *E*. *coli* in the presence of ciprofloxacin. We determined the minimal inhibitory concentration (MIC) of ciprofloxacin on control *E*. *coli* BW25113 cells (carrying the pBAD33 plasmid) as 70 ng/ml. In the presence of low ciprofloxacin concentrations (<40 ng/ml), BW25113 cells carrying the pRIL plasmid grew slower under oxidative stress than did the control cells (Fig 6E). The growth rate of the control cells decreased with the increasing antibiotic concentrations, while the BW25113-RIL cells grew faster under higher ciprofloxacin concentrations (Fig 6E). These results further validated our hypothesis that enhanced tRNA concentrations can help to maintain the translation elongation speed and thus increase resistance to oxidative stress.

## Discussion

All organisms need to adapt environmental oxidative stress. The regulation of translational response is one of the fastest mechanisms whereby cells can guard against sudden environmental stress factors before more specific, persistent mechanisms come into play, such as the *OxyR* and *SoxRS* systems. Although some previous studies have shown that the oxidation of certain aminoacyl-tRNA synthetases can affect the fidelity of translation (reviewed in [48]), a general translational response has remained elusive due to the nearly unchanged transcriptomes and translatomes observed under oxidative stress. Indeed, our results showed that no significant inhibition of protein synthesis could be detected in the *in vitro* translation system in the presence of 0.5 mM H_2_O_2_. This finding suggests that the oxidation of translational machinery components, such as initiation/elongation factors, tRNAs, ribosomes, and mRNAs, are not the direct causal factors in the immediate arrest of protein synthesis *in vivo* during sudden oxidative stress, at least under the stress conditions employed in our study. Of note, this translational arrest was found for nearly all proteins examined, a phenomenon that cannot be caused by the stress translation machinery, which enhances the production of a small group of “leaderless” stress proteins (reviewed in [49]).

The main novelty of this study lays on our discovery of the direct causal factor leading to this rapid and global translation arrest: the enzymatic degradation of tRNA. Our results showed that bacteria respond differently from eukaryotes under oxidative stress in terms of tRNA manipulation. In eukaryotes, tRNAs can be retrotransported into the nucleus and cleaved into halves, or the 3′-CCA tail can be reversibly digested. In contrast, bacteria degrade tRNAs rapidly, without the accumulation of residual tRNA fragments or CCA-lacking tRNAs. These findings indicated that bacteria have a simpler and more direct mechanism of tRNA manipulation than eukaryotes.

Technically, our modified ProVerB MS identification algorithm enabled the efficient identification and quantification of peptides in the full ^15^N metabolic-labeling experiments. We identified ~300–1000 proteins from single *E*. *coli* soluble protein samples, which is at least one order of magnitude higher than previous studies. This improved algorithm provides a general tool for accurate and practical assessments of protein synthesis in prokaryotes and algae, a field, which has lagged far behind eukaryotic studies.

The reduction of tRNA levels under oxidative stress significantly slows down the translation elongation speed. This could be beneficial for prokaryotes because oxidative stress elevates the probability of protein misfolding, and the accumulation of misfolded proteins can be toxic [50,51]. This may be partially compensated by the deceleration of translation elongation, which coordinates protein synthesis and co-translational, domain-wise folding [45]. Further, oxidative stress increases the probability of amino-acid misincorporation (reviewed in [48]), and the slowdown of translation elongation effectively reduces the production rate of mis-synthesized proteins. In sum, tRNA degradation ensures the quality of protein production under oxidative stress, providing a prompt and basal persistence to the bacteria until more specific and effective adaptation mechanism begin to commence.

Interestingly, tRNA supplementation has a dual impact on bacterial fitness. Under normal growth conditions, augmented tRNA levels globally accelerate translation elongation, thereby suppressing translational pausing. This leads to massive protein misfolding and thus impairs fitness [40]. However, under oxidative stress, the tRNA concentration is generally decreased, and the translation elongation is thus largely decelerated. Our results showed that the polysome fraction is remarkably increased under oxidative stress, indicating a much higher ribosome density and thus, an elevated probability of ribosome jamming. Yet, this slow elongation facilitates co-translational folding and effectively rescues the fitness perturbation caused by tRNA augmentation. Higher tRNA concentration also protects cells by decreasing the probability of ribosome jamming that causes the pre-mature dissociation of RNAs from ribosomes [44]. Therefore, the elevated tRNA levels serves to protect against oxidative stress, resulting in enhanced cell growth under mild oxidative stress and survival under harsh oxidative stress.

A natural question is why cells do not increase their tRNA concentrations immediately after oxidative stress. This is unproductive because transcription and complex tRNA processing need a prolonged period of time [52,53]. Results from a previous study showed that the tRNA genes were transcriptionally regulated after 30 min in *E*. *coli* MG1655 cells under stimulus, while no tRNA changes could be detected after 10 min [54]. However, specific oxidation-counteracting systems, such as the *OxyR* and *SoxRS* systems, can exert an effect ~20–30 min after oxidative stress, making tRNA upregulation shortly after oxidative stress less effective. In addition, synthesizing proteins under oxidative stress may elevate the risk for producing oxidized proteins, thereby wasting valuable energy [1]. After cells adapt to oxidative stress, restoring tRNA levels (and thus protein synthesis) is beneficial for further growth. This is exactly what we observed after 90 min of oxidative stress, with many tRNAs reaching higher concentrations than observed under normal conditions (Fig 4, S5 Fig and S6 Fig). In sum, it is an efficient strategy for bacteria to maintain tRNAs at low levels under normal growth conditions and degrade them shortly after encountering oxidative stress.

In addition, we showed that the protective effect of elevated tRNA concentrations also protects bacteria against oxidative stress induced by the antibiotic ciprofloxacin. At a sub-MIC concentration of ciprofloxacin, tRNA-augmented bacteria grew significantly faster than control cells, thereby providing the bacteria with elevated tRNA concentrations a survival advantage in the presence of an antibiotic. Therefore, the global regulation of tRNAs may serve as a general mechanism of antibiotic resistance.

Another question of interest is why eukaryotes tend to reversibly manipulate tRNAs (including the retrotransport of tRNAs into the nucleus and the rapid and reversible cleavage of 3′-CCA) under oxidative stress, while bacteria tend to simply degrade the tRNA irreversibly. A possible answer to this question may relate to the complexity and cost of tRNA production. First, the prokaryotes usually possess ~40–90 tRNA genes in their genomes, while eukaryotic cells express hundreds or thousands of tRNA genes. For example, 506 tRNA genes have been found in human genome hg19, and 12,794 tRNA genes have been found in zebra fish genome zv9, according to the Genomic tRNA Database [55,56]. Complex regulatory information and machineries are needed to control so many tRNA genes separately and efficiently [57]. Second, eukaryotic cells need many more enzymes to process tRNAs than prokaryotes. In *S*. *cerevisiae*, 85 genes are known to process tRNAs to their mature forms and export them to the cytoplasm (reviewed in [58]). In *Arabidopsis thaliana*, 94 genes have been implicated in tRNA modifications [59]. In contrast, *E*. *coli* cells require only 35 enzymes for tRNA modifications [59] and 15 enzymes for tRNA precursor digestion [53]. Third, tRNA processing and transport in eukaryotic cells are spatially localized (reviewed in [58,60]), whereas prokaryotic cells do not have nuclei, and the enzymatic reactions are less spatially organized. Fourth, the transcription speed in prokaryotic cells (~25–65 nt/s in *E*. *coli* [61]) is faster than that in eukaryotes (~18–42 nt/s in yeast [62]). Collectively, these data indicate that regulation in eukaryotic cells involves a much higher energy cost and a much more complex system to produce tRNAs *ab initio* than do prokaryotic cells. Therefore, complex but reversible tRNA manipulation allows eukaryotes to save energy and resume translation elongation after full stress adaptation by avoiding synthesizing and processing tRNAs *ab initio*. In contrast, prokaryotic cells can quickly produce tRNAs and thus tend to maintain a much simpler and irreversible degradation system.

In sum, we found that the global regulation of tRNA mediates rapid adaptation under oxidative stress. This general mechanism provides new insights into the translational responses of bacteria to stress conditions and may offer new views for understanding antibiotic resistance in bacteria.

## Materials and Methods

### Bacterial strains and plasmids

*E*. *coli* BL21(DE3) and wild-type BW25113 strains were used in this study. To increase the tRNA concentrations, bacteria were transformed with the pRIL plasmid that was isolated from the *E*. *coli* BL21(DE3)-CodonPlus-RIL strain (Stratagene). This plasmid confers chloramphenicol resistance and encodes the tRNA genes *argU*, *ileY*, and *leuW* that recognize AGA, AGG, AUA, and CUA codons. In this study, these three tRNAs were designated as 5-Arg(TCT), 17-Ile(GAT), and 22-Leu(TAG), respectively (Fig 4). We used the empty vector pBAD33, which also confers chloramphenicol resistance as a control for the pRIL plasmid.

### Growth curve measurement

To accurately measure bacterial growth rates in batches, we assembled in house a fully automatic device to measure ODs. Bacteria were grown in rectangular, sterilized cuvettes, referred to here as “measuring units.” A narrow 635-nm laser beam was used as a light source, and the penetrating light was measured by a digital illuminometer connected to computer (S7A Fig). Optical densities were calculated according to the Beer-Lambert Law. Data were recorded automatically via the computer with a minimal interval of 10 s. During cell growth, measurements were continuously obtained without interfering with the shaker, which prevented temperature fluctuations caused by opening and closing the lid. Six such measuring units were fixed on a 30° slope and installed in an orbital shaker for culturing purposes (S7B Fig). No sedimentation of *E*. *coli* cells was noted after 12 h of culturing with shaking at 200 rpm.

We validated the measurements obtained with our in-house constructed device by comparison with the Genesys 10S UV-Vis spectrophotometer (Thermo). Our device yielded comparable results compared to the Genesys 10S UV-Vis spectrophotometer between an OD range of ~0–0.45, with a correlation coefficient approaching 1 (S7C Fig).

For each growth curve measurement, the *E*. *coli* cells were pre-cultured in LB medium at 37°C overnight and then collected by centrifugation, resuspended, and added in M9 minimal medium at a 1:1000 dilution, yielding an initial OD of <0.005. The measurements were performed within the linear range of our device (OD = 0~0.45).

### Cell viability assessments

Cell viabilities were determined via propidium iodide (PI) staining [63,64]. Briefly, *E*. *coli* cells were harvested at OD = 0.05 by centrifugation and diluted in phosphate-buffered saline (PBS) to ~10^6^ cells/mL. A 100-μl suspension was supplemented with PI (Sigma) to a final concentration of 10 μg/mL and incubated for 30 min at 4°C. After centrifugation at 12,000 × *g* for 2 min at 4°C, the cell pellet was resuspended in 1 mL of PBS twice to remove unbound PI. Flow cytometry was performed using a C6 flow cytometer (BD Accuri), using previously described settings [64].

### Metabolic labeling with ^15^N

*E*. *coli* BL21(DE3) cells were cultivated in M9 minimal medium with 0.2% glucose at 37°C in an orbital shaker until the OD reached 0.6, and cells were harvested at room temperature by centrifugation at 5000 × *g* and washed twice with M9 minimal medium to remove any remaining ^14^NH_4_Cl. The cells were then resuspended in an equal volume of ^15^NH_4_Cl-containing M9 medium. To generate oxidative stress, H_2_O_2_ (Sigma) was also added to a final concentration of 0.5 mM. Cells were further cultured, and samples were obtained at the indicated time points.

### Mass spectrometry of proteins

Harvested cells were washed with PB) and resuspended in urea lysis buffer (8 M urea, 2 M thiourea, 4% CHAPS, 15 mM DTT, and 1 mM PMSF). Cell lysates were sonicated in an ice bath for 5 min and centrifuged 15,000 × *g* for 15 min at 4°C to remove cell debris. In-gel digestion was performed, as described previously [65]. Briefly, 40 μg of protein was resolved on a 12% SDS-PAGE gel. The gel bands were cut into 4 slices and in-gel digestion was performed with MS-grade trypsin (Promega). MS was performed on an LTQ-Orbitrap mass spectrometer (Thermo Scientific), equipped with a nanospray source as described previously [66].

### Protein identification and quantification

dta-format files were generated using BioWorks software, version 3.3.1 (Thermo-Finnigan, San Jose, CA) and searched against real and decoy *E*. *coli* reference protein databases, using a modified version of ProVerB software [36]. We modified the ProVerB program while considering the following terms: 1) the amino acid table contains 40 types of amino acids (20 ^15^N-labeled amino acids and 20 ^14^N (unlabeled) amino acids), 2) the theoretical peptide database contains ^15^N peptides and ^14^N peptides, 3) candidate peptides for each spectrum were selected from the theoretical *E*. *coli* peptide database according to precursor ion mass, and 4) all candidates were assessed by determining ProVerB scoring functions. The following search criteria were employed: full tryptic specificity was required, 2 missed cleavages were allowed, and carbamidomethylation was set as a fixed modification, whereas oxidation (M) was considered a variable modification. Precursor ion mass tolerances were set at 10 ppm for all MS spectra acquired in an LTQ-Orbitrap mass analyzer, and the fragment ion mass tolerance was 0.5 Da for all MS^2^ spectra acquired. All top peptides from each spectrum were filtered using a FDR of ≤0.01, and the protein group was built as the set of all filtered peptides.

To quantify the ^14^N/^15^N ratio for each protein, all filtered peptides and grouped proteins were converted to the dtaselect (version 1.9)-output format. The raw files from the LTQ-Orbitrap instrument were converted to MS1 format using RawXtract software, version 1.93. Census software (version 1.7) was used to the calculate area ratio, defined as the ratio of the ^14^N peptide peak area over the ^15^N peptide peak area in a given chromatogram.

### Protein synthesis and half-life calculations

After cells were transferred from ^14^N medium to ^15^N medium (time point *t*_0_), the ^15^N-labeled proteins began to be synthesized. The ^14^N protein decay can be described as shown in Eq 1

$$N_{14}(t)=N_{0}\cdot e^{-k_{\text{deg}}(t-t_{0})}$$ (1)

where *N*_0_ is the amount of protein at *t*_0_, *N*_14_(*t*) is the amount of ^14^N protein at time *t*, and *k*_deg_ is the degradation rate constant. Given that the proteome is generally in steady state during exponential growth under normal and oxidative-stress conditions (see [24,28,67] and data in Fig 2A from this study), we have *N*_0_ = *N*_14_(*t*)+*N*_15_(*t*) and *k*_deg_ = *k*_syn_, where *k*_syn_ is the synthesis rate constant. Therefore, Eq 1 can be reformulated as follows:

$$N_{14}(t)=[N_{14}(t)+N_{15}(t)]\cdot e^{-k_{syn}(t-t_{0})}$$

Thus,

$$\frac{N_{14}(t)+N_{15}(t)}{N_{14}(t)}=e^{k_{syn}(t-t_{0})}$$ (2)

Let *R* be the ratio between the ^14^N and ^15^N proteins: $R=\frac{N_{14}(t)}{N_{15}(t)}$, which is the value measured by MS. Eq 2 can be then rewritten as follows:

$$1+\frac{1}{R}=e^{k_{syn}(t-t_{0})}$$ (3)

By letting

$$Y=\text{ln}\left(1+\frac{1}{R}\right)$$ (4)

and substitute Y into Eq 3, we derive the equation:

$$Y=k_{syn}(t-t_{0})$$ (5)

Therefore,

$$\frac{dY}{dt}=k_{syn}$$ (6)

According to Eq 6, *k*_syn_ can be fitted using the dataset of *Y* and *t*. Rapid protein synthesis is represented by a high *k*_syn_ value, which corresponds to a high slope for *dY/dt*.

The half-life time of the protein is then calculated as

$$T=\frac{\text{ln}2}{k_{syn}}$$ (7)

### Cell-free expression

The S30 T7 High-Yield Cell-Free Protein Expression System (Promega) was used for cell-free expression tests under oxidative stress, according to the manufacturer’s recommended protocol. The *E*. *coli* SufI gene inserted into pET-28b plasmid with C-terminal His_6_ tag was expressed according to the manufacturer’s recommended protocol. The reactions were incubated for 20 min, stopped, and then assessed by western blot analysis.

### Isolation of detergent-insoluble proteins

*E*. *coli* cells were harvested at OD_635nm_ = 0.05. The detergent-insoluble proteins (aggregated proteins) were isolated as described in our previous report [40].

### Polysome profiles

Polysome profiles were generated as described previously [44]. Briefly, debris-free cell lysates were layered onto 30 mL 15–40% sucrose gradients and centrifuged at 30,000 rpm for 6 h in an SW32Ti rotor (Beckman). Gradients were slowly pumped out and detected at 254 nm with a UV spectrophotometer.

### Spike-in preparation and total RNA extraction

Spike-in RNA (5′- CGAAAUUAAUACGACUCACUAUAGGGGAAUUGUGAGCGGAUAACUGACUGACUGACUAAAUAAUUUUGUUUAACUUUAAGAAGGAGAUAUACCA-3′) was produced using the RiboMAX Large-Scale RNA Production System-T7 (Promega) using a synthetic DNA template with a T7 promoter. The spike-in was purified using TRIzol (Ambion), according to the manual and quantified using a NanoDrop 2000 Spectrophotometer (Thermo).

Cells were harvested by centrifugation 8000 × *g* for 15 min at 4°C and resuspended in 1 mL TRIzol (Ambion). Spike-in RNA was added to each sample at 500 ng/(OD•mL). RNA extraction was then performed according to the product manual, with the modification that the RNA was precipitated overnight in isopropanol at -20°C. The remaining DNA was removed by DNaseI treatment (Fermentas), according to the product manual.

### qRT-PCR analysis of tRNAs

tRNA deacylation was performed as described previously [68], except that deacylation was performed at a pH of 10. A poly(A) tail was added to the deacylated 3′-CCA terminus using *E*. *coli* poly(A) polymerase (New England BioLabs), according to the product manual. Prior to reverse transcription, polyadenylated tRNA were denatured for 5 min with a TG primer (5′-T_15_GG-3′; 100 μM) and dNTPs (25 mM each) at 80°C and then chilled on ice. Reverse transcription was performed with RevertAid Premium Reverse Transcriptase (Fermentas), according to the product manual.

Specific primers for amplifying 39 *E*. *coli* tRNAs were designed using Primer-Premier 5 software and are listed in S3 Table. The specificity of these primers was verified both by *in silico* analysis (NCBI Primer-Blast) and melting-curve analysis after qPCR amplification. qPCR was then performed with each of the 39 tRNA-specific primer sets and SsoFast Evagreen Supermix (Bio-Rad) on a Bio-Rad MiniOpticon Real-Time PCR system (Bio-Rad), according to the manufacturer’s instructions.

### Next-generation sequencing of tRNAs and data processing

After reverse transcribing tRNA to cDNA, second-strand synthesis was performed using RNase H, DNA Polymerase I, and T4 DNA Polymerase (Fermentas) according to the manufacturer’s recommended procedure. The double-stranded cDNA was then purified using a HiPure PCR Kit (Magen). A sequencing library was constructed using the NEBNext Ultra DNA Library Prep Master Mix Set for Illumina (NEB). Next-generation sequencing was performed on an Illumina HiSeq-2000 sequencer for 100 cycles. High-quality reads passing the Illumina filter were kept for subsequent data analysis. The sequencing dataset was deposited in the Gene Expression Omnibus database under Accession Number GSE62995.

Adapter sequences were trimmed from the reads. The reads were aligned to tRNA reference sequences using the modified Smith–Waterman algorithm that considered modified nucleotides matching any nucleotide [43]. A maximum of 3 errors (including mismatches, insertions, and deletions) was allowed in the alignments, and the minimal alignment length was set to 25 bp. The number of aligned tRNA reads were then normalized using the spike-in reads.

## Supporting Information

S1 Fig Identification of protein peptides. The representative MS2 spectra of FESEVYILSK of EF-Tu (A) and GGVIPGEYIPAVDK of EF-G (B) were identified by using modified ProVerB algorithm and shown with b and y ions indicated with cyan lines and red lines.
(TIF)

S2 Fig Quantification of the ^14^N/^15^N ratio of two peptides (FESEVYILSK and GGVIPGEYIPAVDK) in normal condition and oxidative stress as examples. Samples were taken 60min after switching to ^15^N M9 medium. Y-axes indicates the MS peaks intensity of the protein peptides. Signals of ^14^N and ^15^N protein peptides were indicated by blue and red lines. The relative abundance ratio of each ^14^N and ^15^N protein corresponds to the ratio of their peak area. Quantification of two protein peptides in normal condition (A) and under oxidative stress (B).
(TIF)

S3 Fig Phylogenetic tree of spike-in RNA and *E*. *coli* tRNA sequences, calculated using DNASTAR software. (TIF)

S4 Fig Reproducibility tests of three biological replicates of *E*.*coli* tRNA under normal condition using next-generation sequencing (A) and qRT-PCR (B). In all panels, the data were normalized using spike-in RNA. The read count ratio (A) is the tRNAs read count/spike-in RNA read count ration. Raw read counts were listed in S1B Table. ΔC_T_ (B) indicates spike-in RNA C_T_—tRNA C_T_. In all panels, the Pearson and Spearman correlation coefficients (r_p_ and r_s_) were indicated, respectively.
(TIF)

S5 Fig tRNA quantification using random hexamer to prime reverse transcription. The tRNAs extracted 15min (left panel) and 90min (right panel) after oxidative stress were tested, respectively. Ct values were normalized using spike-in RNA. Data are shown in mean ± SD.
(TIF)

S6 Fig tRNA levels quantified using next-generation sequencing in wild-type *E*. *coli* BW25113 strain, 15min and 90min after oxidative stress, respectively. Please refer to Fig 4 for details.
(TIF)

S7 Fig In-house made device to measure the bacterial growth curve continuously and automatically. (A) Design of one measuring unit of this device. (B) The device with 6 measuring units installed in a shaker. The Analogue-Digital Converters (ADCs) were placed outside of the shaker and connected to a computer to record data. When operating, the whole system was kept away from ambient light. (C) Comparison of the OD_635nm_ measured by our in-house made device and the Genesys 10S UV-Vis spectrophotometer. Pearson and Spearman correlation coefficients (*r*_*p*_ and *r*_*s*_) were indicated in the diagrams, respectively.
(TIF)

S1 Table ^14^N/^15^N protein ratio of all identified proteins after the application of H_2_O_2_ under normal condition and oxidative stress, respectively. Data obtained in *E*. *coli* BL21(DE3) and BW25113 strains are included.
(XLS)

S2 Table The read counts of all tRNAs and spike-in RNA: (A) under normal condition and oxidative stresses (15min, 30min and 90min, *E*. *coli* BL21(DE3) and BW25113 strains); (B) three independent biological replicates of normal condition. (XLSX)

S3 Table Specific qPCR primers for 37 *E*. *coli* tRNAs. (DOCX)
