# Supplementary figures and images for: Transfer RNAs Mediate the Rapid Adaptation of Escherichia coli to Oxidative Stress
Source: PLoS Genet. 2015 Jun 19;11(6):e1005302. doi: 10.1371/journal.pgen.1005302 (PMC4474833; doi:10.1371/journal.pgen.1005302)

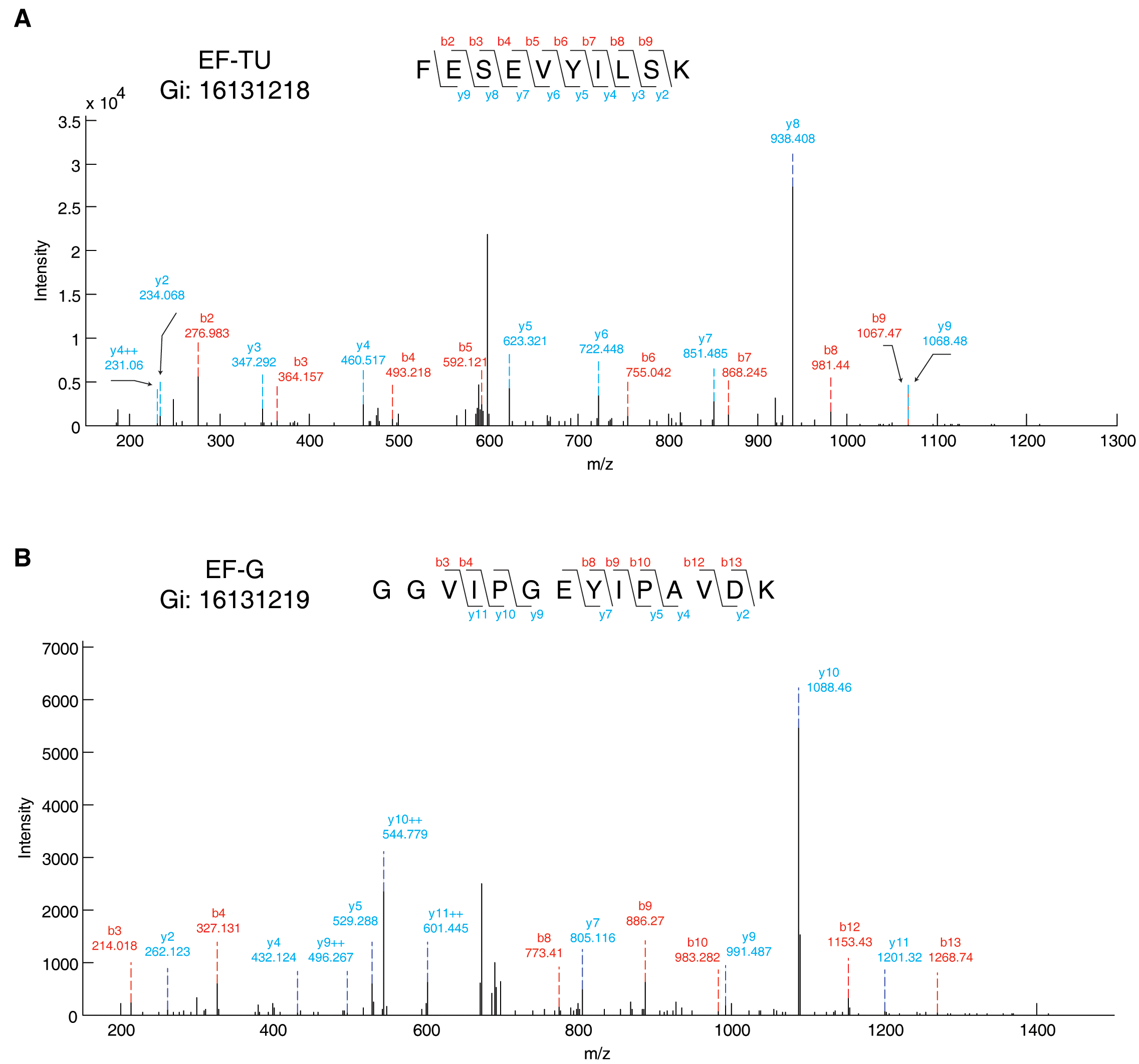

Supplement: S1 Fig — The representative MS2 spectra of FESEVYILSK of EF-Tu (A) and GGVIPGEYIPAVDK of EF-G (B) were identified by using modified ProVerB algorithm and shown with b and y ions indicated with cyan lines and red lines. (TIF) [file pgen.1005302.s001.tif]

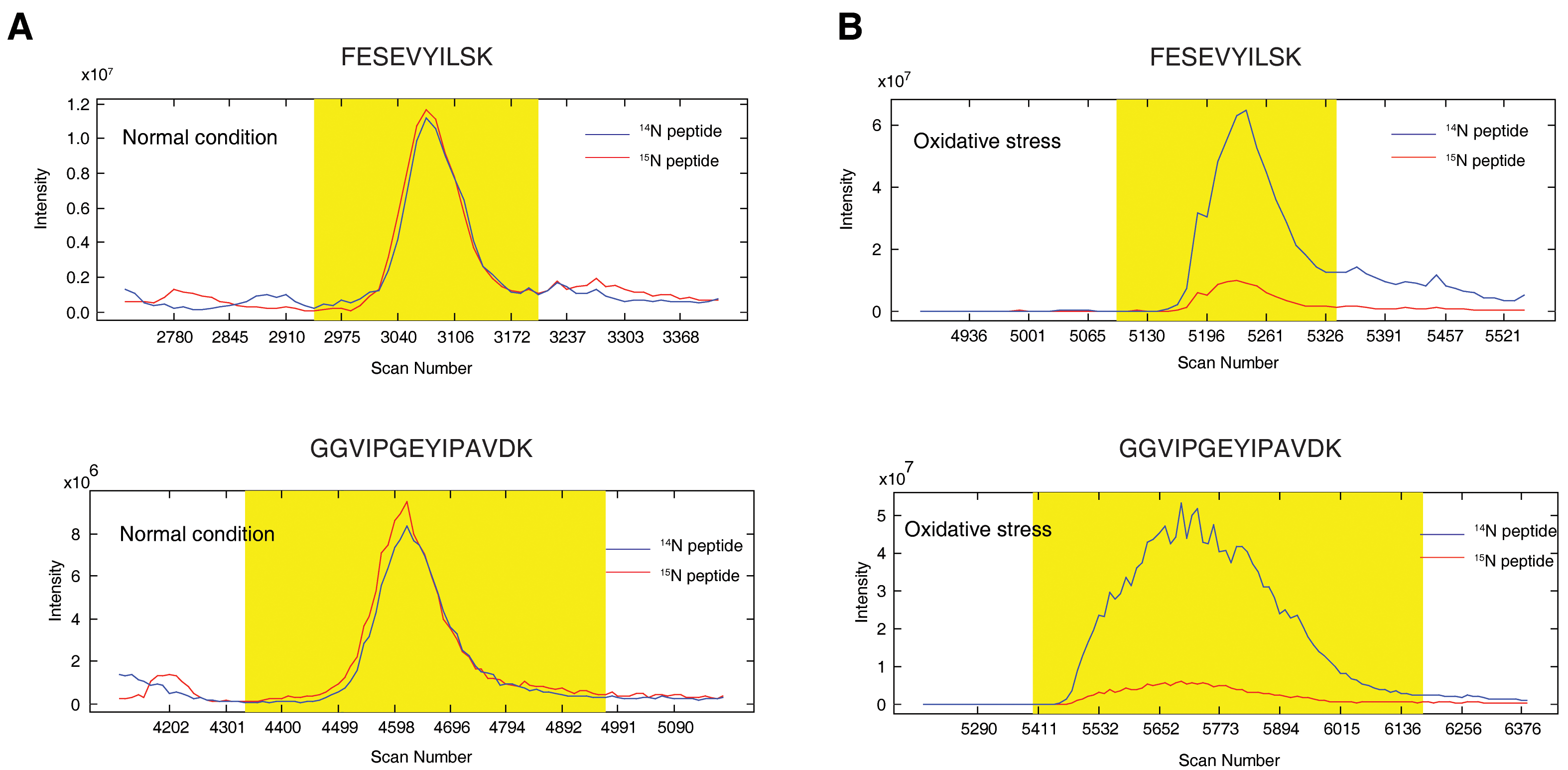

Supplement: S2 Fig — Samples were taken 60min after switching to 15N M9 medium. Y-axes indicates the MS peaks intensity of the protein peptides. Signals of 14N and 15N protein peptides were indicated by blue and red lines. The relative abundance ratio of each 14N and 15N protein corresponds to the ratio of their peak area. Quantification of two protein peptides in normal condition (A) and under oxidative stress (B). (TIF) [file pgen.1005302.s002.tif]

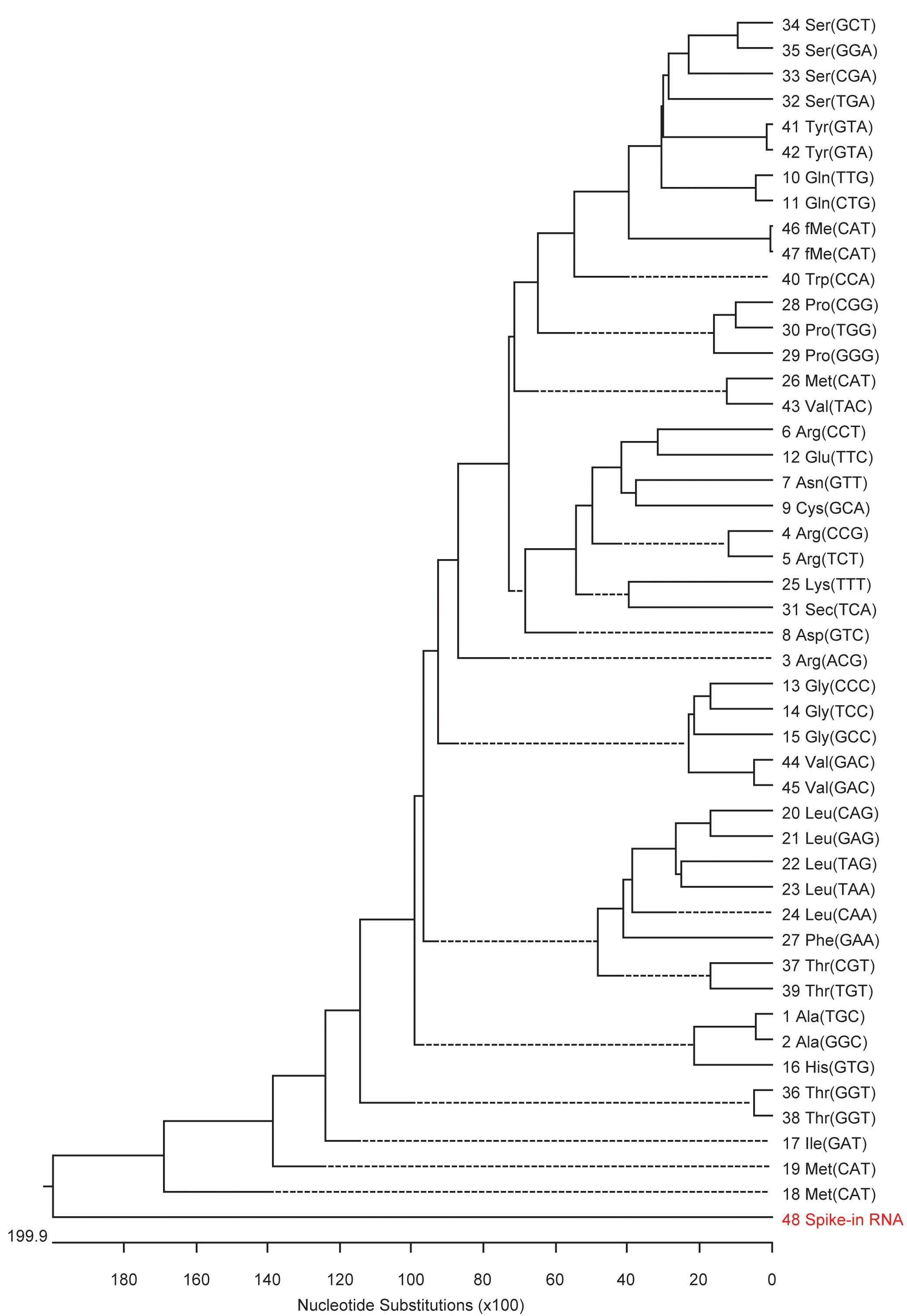

Supplement: S3 Fig — (TIF) [file pgen.1005302.s003.tif]

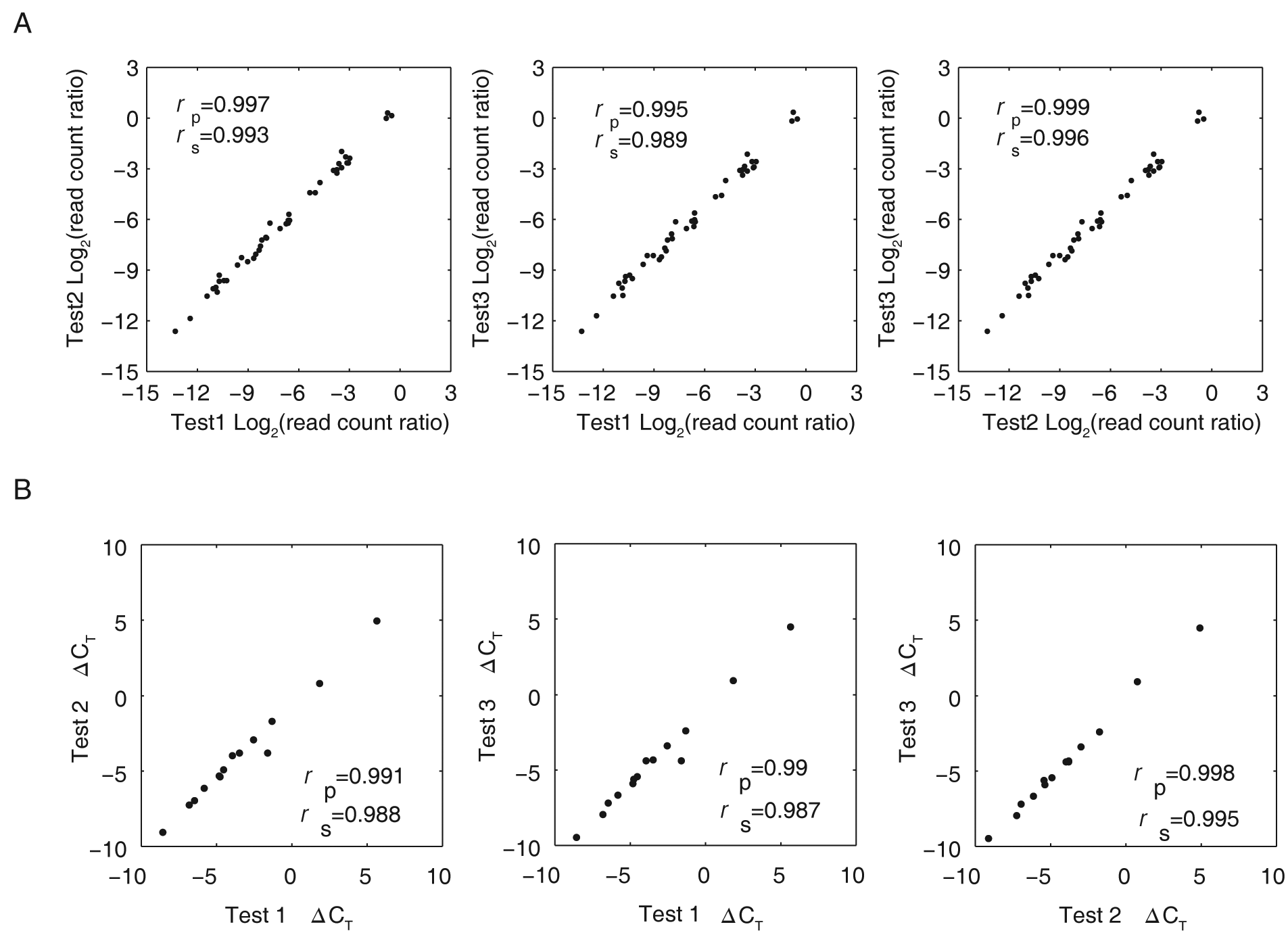

Supplement: S4 Fig — In all panels, the data were normalized using spike-in RNA. The read count ratio (A) is the tRNAs read count/spike-in RNA read count ration. Raw read counts were listed in S1B Table. ΔCT (B) indicates spike-in RNA CT—tRNA CT. In all panels, the Pearson and Spearman correlation coefficients (rp and rs) were indicated, respectively. (TIF) [file pgen.1005302.s004.tif]

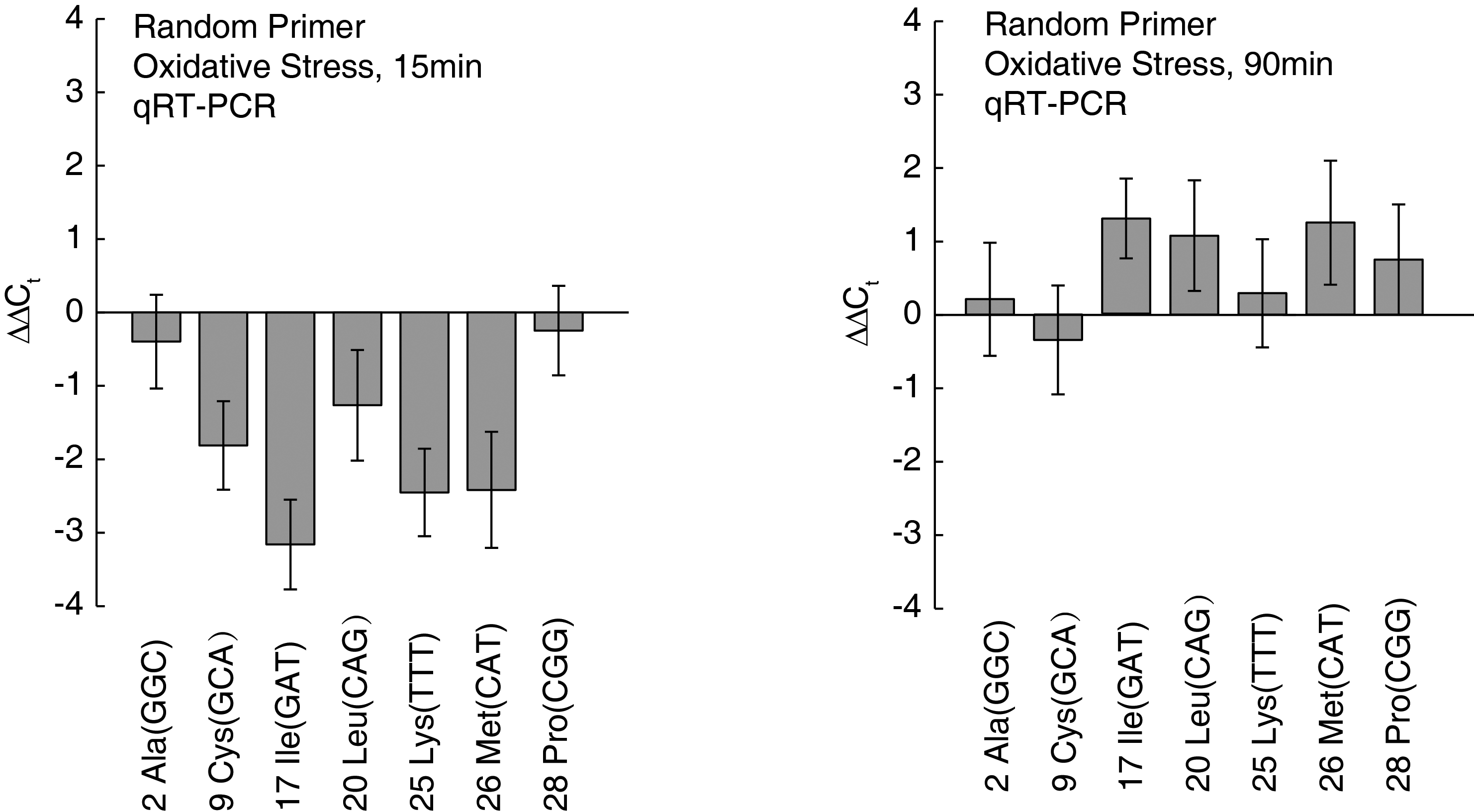

Supplement: S5 Fig — The tRNAs extracted 15min (left panel) and 90min (right panel) after oxidative stress were tested, respectively. Ct values were normalized using spike-in RNA. Data are shown in mean ± SD. (TIF) [file pgen.1005302.s005.tif]

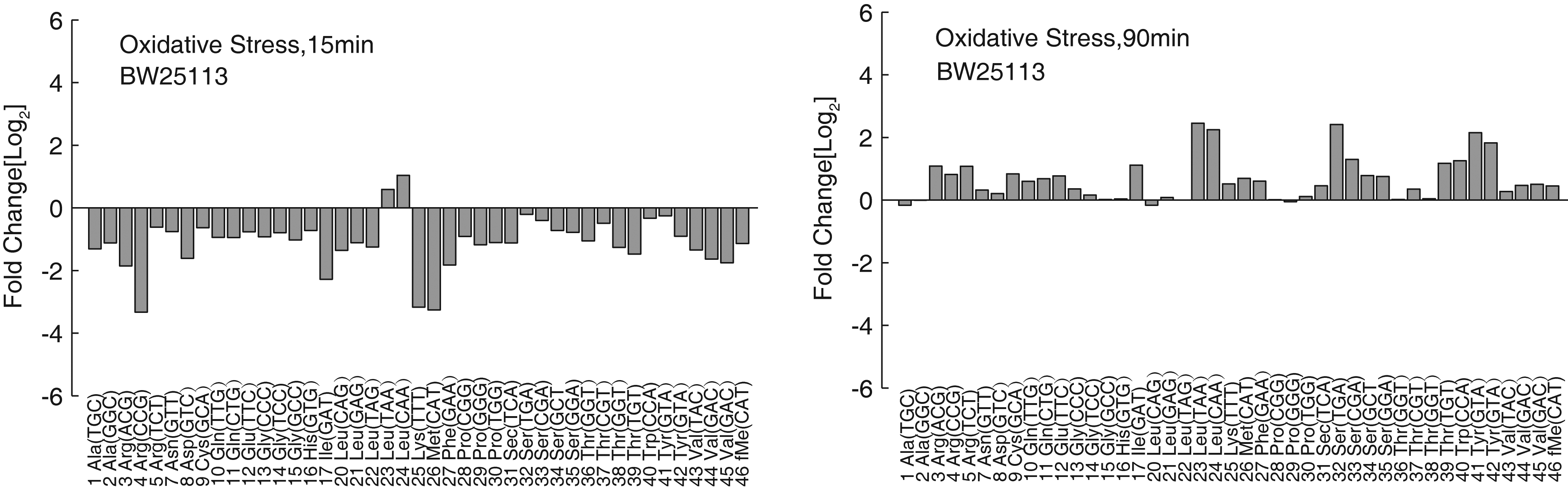

Supplement: S6 Fig — Please refer to Fig 4 for details. (TIF) [file pgen.1005302.s006.tif]

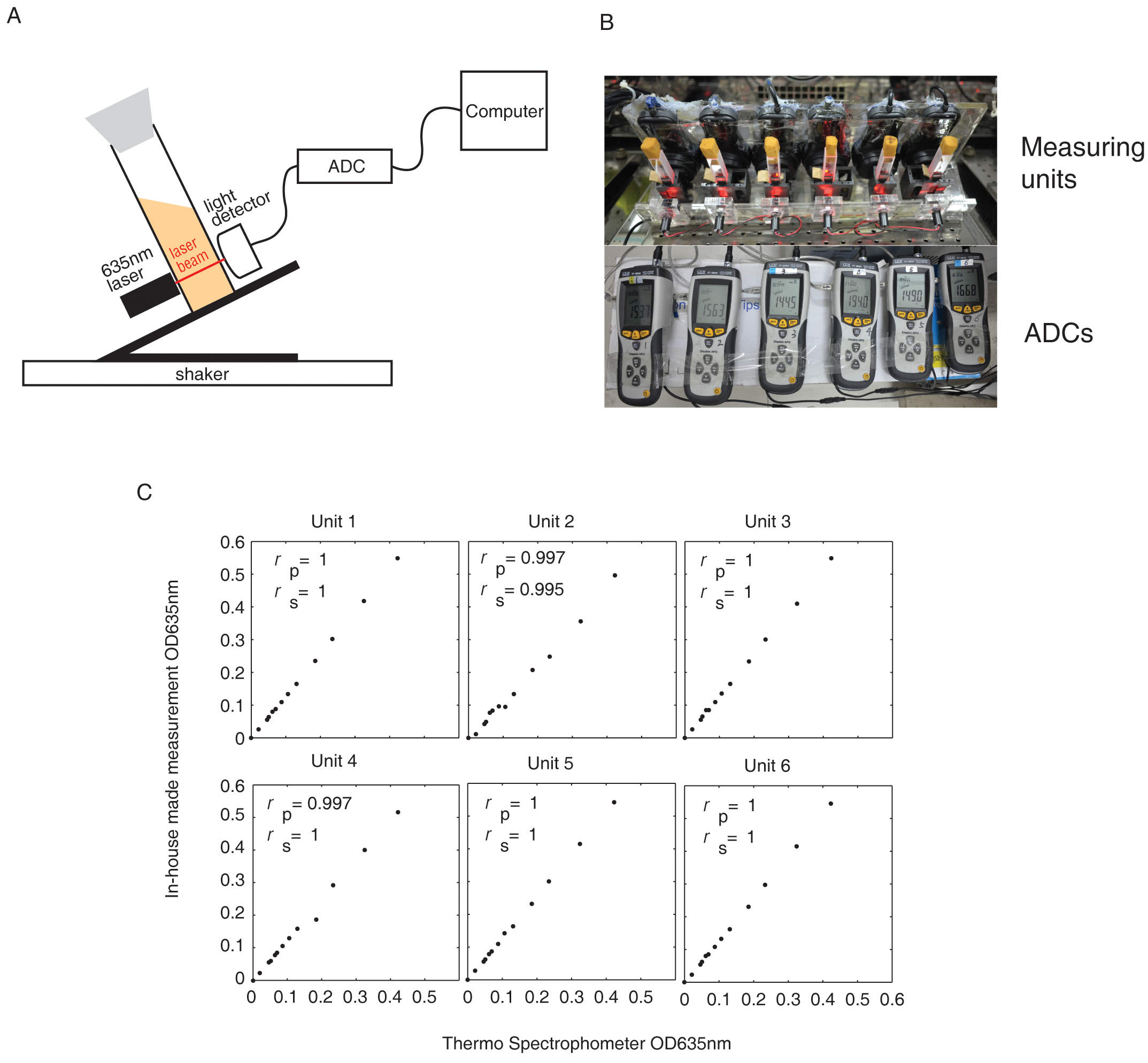

Supplement: S7 Fig — (A) Design of one measuring unit of this device. (B) The device with 6 measuring units installed in a shaker. The Analogue-Digital Converters (ADCs) were placed outside of the shaker and connected to a computer to record data. When operating, the whole system was kept away from ambient light. (C) Comparison of the OD635nm measured by our in-house made device and the Genesys 10S UV-Vis spectrophotometer. Pearson and Spearman correlation coefficients (r p and r s) were indicated in the diagrams, respectively. (TIF) [file pgen.1005302.s007.tif]
