# Supplementary material for: Transfer RNAs Mediate the Rapid Adaptation of Escherichia coli to Oxidative Stress
Source: PLoS Genet. 2015 Jun 19;11(6):e1005302. doi: 10.1371/journal.pgen.1005302 (PMC4474833; doi:10.1371/journal.pgen.1005302)
Supplement: S3 Table — (DOCX) [file pgen.1005302.s010.docx]

**Table S3:** Specific qPCR primers for 37 *E. coli* tRNAs

| NO. | tRNA | Forward Primer | Reverse Primer |
| --- | --- | --- | --- |
| 1 | 2;Ala;(GGC); | CAGCTGGGAGAGCGCT | ACCGCTGACCTCTTGCA |
| 2 | 1;Ala;(TGC); | TCAGCTGGGAGAGCGCC | GAACCGCAGACCTCCTGCG |
| 3 | 3;Arg;(ACG); | CAGCTGGATAGAGTACTCGG | GGGAGGATTCGAACCTCC |
| 4 | 4;Arg;(CCG); | CTGGATAGAGCGCTGCC | CGACAGGATTCGAACCTGA |
| 5 | 7;Asn;(GTT); | GTAGTTCAGTCGGTAGAACGG | CTCTGACTGGACTCGAACCA |
| 6 | 8;Asp;(GTC); | GGTAGTTCAGTCGGTTAGAATAC | GGGACGGGACTCGAAC |
| 7 | 9;Cys;(GCA); | GCGTTAACAAAGCGGTTATGTAGC | TGGAGGCGCGTTCCGGAGTCG |
| 8 | 11;Gln;(CTG); | CGGTAAGGCACCGGATTC | GGTACGAGGATTCGAACCTC |
| 9 | 10;Gln;(TTG); | CGGTAAGGCACCGGTTTT | GGTACCTGGATTCGAACCAG |
| 10 | 12;Glu;(TTC); | CTTCGTCTAGAGGCCCAGG | CCCTAGGGGATTCGAACC |
| 11 | 13;Gly;(CCC); | GCGTAGTTCAATGGTAGAACG | GAAGGGAATCGAACCCTCG |
| 12 | 15;Gly;(GCC); | GAATAGCTCAGTTGGTAGAGCAC | GAGACTCGAACTCGCGACC |
| 13 | 14;Gly;(TCC); | GCATCGTATAATGGCTATTACC | GCAGCGGGAATCGAACC |
| 14 | 16;His;(GTG); | GCTATAGCTCAGTTGGTAGAGC | TGGGATTCGAACCCACG |
| 15 | 17;Ile;(GAT); | CCCTTAGCTCAGTGGTTAGAGC | GTGGCCCCTGCTGGACTTGAACC |
| 16 | 20;Leu;(CAG); | GGAATTGGTAGACGCGCTAGC | TGGTGCGAGGGGGGGGAC |
| 17 | 21;Leu;(GAG); | TAGACACGCTACCTTG | TGGTACCGAGGACG |
| 18 | 22;Leu;(TAG); | GCGGGAGTGGCGAAATTGG | TGGTGCGGGAGGCGAGAC |
| 19 | 24;Leu;(CAA); | GAAGTGGCGAAATCG | GGCACGTATTTCTACG |
| 20 | 23;Leu;(TAA); | CGGATGGTGGAATCG | CGCACAGCGCGAAC |
| 21 | 25;Lys;(TTT); | CGTTAGCTCAGTTGGTAGAGCAG | CGTGCAGGATTCGAACCTG |
| 22 | 46;fMe;(CAT); | CGCGGGGTAGCTCAGTTGG | GTTGCGGGGACGGGATTCG |
| 23 | 27;Phe;(GAA); | GCCCGGATAGCTCAG | CCGGACTCGGAATCG |
| 24 | 28;Pro;(CGG); | CCTGGTAGCGCACTTCG | GTGATAGAGGATTCGAACCTC |
| 25 | 29;Pro;(GGG); | CCTGGTAGCGCACCGTC | GAGAGGATTTGAACCTCCGAC |
| 26 | 30;Pro;(TGG); | GCAGCTTGGTAGCGCAAC | TTCGAACCTCCGACCCAC |
| 27 | 31;Sec;(TCA); | CTCCGGTGAGGCGGC | ACAGGAGTCGAACCTGCCC |
| 28 | 33;Ser;(CGA); | CGGCTGAACGGACCG | GATTTGAACCCCCGGTAGAG |
| 29 | 35;Ser;(GGA); | TGTCCGAGTGGCTGAAGGAG | GGATTCGAACCCCCGATAC |
| 30 | 34;Ser;(GCT); | AGAGGCTGAAGGCGCTCC | CGAACCCCGGATGCAGC |
| 31 | 32;Ser;(TGA); | TGGCCGAGCGGTTGAAG | CGCAGAGATTCGAACTCTGG |
| 32 | 37;Thr;(CGT); | ATAGCTCAGTTGGTAGAGCAGCG | CCGATAATAGGAGTCGAACCTAC |
| 33 | 39;Thr;(TGT); | CTTAGCTCAGTAGGTAGAGCAACTG | GACTACCGGAATCGAACTGG |
| 34 | 40;Trp;(CCA); | GCGTAGTTCAATTGGTAGAGCACC | TGGCAGGGGCGGAGAGACTC |
| 35 | 42;Tyr;(GTA); | CCAAAGGGAGCAGAC | GGGAAGGATTCGAACC |
| 36 | 44;Val;(GAC); | CGTTCATAGCTCAGTTGGTTAGAG | CGTTCAATTGGACTCGAACCA |
| 37 | 43;Val;(TAC); | GATTAGCTCAGCTGGGAGAGC | GGTGATGACGGGATCGAAC |
